# Digital Literacy in the Informal Economy of Ghana: Life-long Learning and Extending Working Lives of Older Persons in Post-Covid-19 Era

**DOI:** 10.1007/s10823-024-09514-9

**Published:** 2024-08-19

**Authors:** Samuel Ampadu Oteng, Esmeranda Manful, Jacob Oppong Nkansah

**Affiliations:** 1 School of Graduate Studies, Lingnan University, 8 Castle Peak Road, Tuen Mun, New Territories, Hong Kong; 2 Department of Sociology and Social Work, Kwame Nkrumah University of Science and Technology, Kumasi, Ghana

**Keywords:** Digital literacy, Older workers, Informal economy, COVID-19, Life-long learning, Ghana

## Abstract

The COVID-19 pandemic has underscored the growing importance of digital technologies for economic resilience, especially for vulnerable groups like older workers in the informal sector. However, barriers to access and digital literacy create challenges alongside potential opportunities, particularly in less developed countries such as Ghana. Using older adults over 50 years engaged in informal work in Kumasi’s Central Business District in Ghana as a case, this paper explores older informal workers’ use of digital technologies in Ghana during the pandemic. Findings suggest that older informal workers relied heavily on their mobile phones as the only critical technological tool to sustain their businesses during the COVID-19 pandemic. However, the findings also reveal critical gaps in skills, training, and support, alongside resourcefulness in leveraging digital tools for business continuity. Key policy implications include expanding mobile-centric digital literacy programs, addressing infrastructure divides, and integrating capacity building into social protection. The paper contributes insights on strengthening lifelong learning and extending the working lives of older persons in the informal sector in the post-COVID era.

## Introduction

The informal sector represents a significant portion of global employment, accounting for 90% of jobs in low-income countries, 67% in middle-income countries, and 18% in high-income countries (Bonnet et al., 2019). This sector encompasses small-scale, self-employed, or family-based enterprises that operate outside formal regulatory frameworks (ILO, 2015). At the same time, the global population is rapidly ageing, as the number of older persons is projected to grow from 727 million in 2020 to 1.5 billion in 2050 (UN, 2020). Moreover, many older informal workers express a willingness to continue working for as long as they are able, highlighting their motivation for sustained labor force participation into later life (Oteng et al., 2022). Given that the informal sector accounts for most employment globally, and the ageing trend affecting the entire workforce, it is reasonable to conclude that a substantial portion of older workers are engaged in informal economic activities (Petrova, 2019). Before the COVID-19 pandemic, older informal workers already faced distinct challenges, such as limited social protection, lower incomes, and poorer working conditions compared to their formal sector counterparts (ILO, 2015, 2020). Reduced productivity and barriers to training have further hampered their ability to adapt to changing economic conditions, leading to greater financial insecurity and employment difficulties as they age (ILO, 2018; Phillipson, 2019). The pandemic exacerbated these issues, with 80% of older informal workers reporting reduced working hours and income since the crisis began (ILO, 2020; Sein, 2020; Sixsmith et al., 2022). Therefore, the economic disruptions have disproportionately affected this vulnerable group, worsening the disparities they face. Yet, the COVID-19 pandemic has underscored the crucial importance of digital access and skills, especially for vulnerable groups like older informal workers (Hecker et al., 2021; Ratten, 2020). Specifically in less developed countries, studies have shown that up to 70% of older informal workers have limited experience using digital technologies for their work, which has posed challenges during the shift to remote and contactless work (ILO, 2018, 2020).

The digital divide between older and younger adults is well-documented, with older adults generally having lower levels of internet use and digital literacy, especially in less developed countries (Hunsaker & Hargittai, 2018). For example, in Ghana, the digital landscape is evolving rapidly, yet older adults often face significant barriers, including lack of access, limited digital skills, and low confidence, to incorporating digital literacy into their lives and livelihoods (Dovie, 2018; 2019). This divide is further exacerbated by perceived ageism, which can discourage older adults from engaging with digital technologies (Meliou et al., 2019). The informal sector, on the other hand, presents additional challenges for older workers in acquiring digital literacy in Ghana (Dovie, 2018; Oteng et al., 2024). Workers in this sector often lack access to formal training and resources that could help them develop digital skills. Financial constraints and the absence of institutional support further limit their ability to engage with digital technologies that have become ubiquitous in informal trade and entrepreneurship (Neumeyer et al., 2020). These knowledge gaps, accessibility, and affordability barriers inhibit the effective utilization of technologies that could enable remote work and learning (Hecker & Briggs, 2021; Muro et al., 2017). With mobility restrictions and reduced face-to-face engagement, technology became even more essential for economic and social participation during the pandemic (Hecker & Briggs, 2021; ILO, 2020). However, longstanding gaps in digital literacy and infrastructure access faced by many older adults posed significant challenges in navigating basic needs through digital means, from marketing goods to accessing healthcare in Ghana (Anakpo & Mishi, 2021; Dovie, 2019). The acceleration of technology adoption and digitization in informal sector activities during the COVID-19 pandemic has further exacerbated these challenges for older workers (Dovie, 2019). Lacking digital proficiency, many informal workers, including older adults were less resilient to the disruptions caused by the pandemic compared to their tech-savvy younger counterparts (Bartik et al., 2020; Budnitz & Tranos, 2022). The pandemic has highlighted the urgent need to bridge these digital divides through targeted training and infrastructure development initiatives, to support the economic and social inclusion of older informal workers (Erlanitasari et al., 2020; Hecker & Loprest, 2019).

Despite the valuable insights provided by existing studies, there remains a critical gap in research specifically focusing on the unique experiences and challenges faced by older individuals engaged in informal economic activities. Much of the current literature on ageing workers tend to address older adults more broadly, often overlooking the distinct circumstances of those operating in the informal sector. Notably, recent work by Oteng et al. (2024) sheds light on the damaging effects of stereotypes and discrimination regarding older informal workers’ ability and willingness to utilize digital technologies. However, these ageist assumptions and attitudes fail to account for the significant number of older informal workers who are both capable and eager to develop essential digital skills (Papadopoulos et al., 2020). The exclusion of this demographic from lifelong learning opportunities is not only a denial of their fundamental right to education, but also prevents the realization of other crucial rights, such as the entitlement to decent work, health, and overall wellbeing (Cebola et al., 2023). This oversight severely limits the understanding of the unique challenges faced by older informal workers in acquiring digital competencies, which in turn impacts their productivity, and incomes, and exacerbates social isolation as economic activities rapidly digitalize (Dovie et al., 2019; Asongu et al., 2018). There is also a need for empirical studies that evaluate the effectiveness of digital literacy programs tailored to older informal workers. Current literature lacks evidence on the impact of such programs on improving digital skills and overall well-being. Owing to this, this study aims to develop an in-depth understanding of the specific issues faced by older informal workers in Ghana, their technology use during the pandemic, knowledge gaps inhibiting adoption, and potential training and policy solutions. The overarching objective is to generate insights that can inform targeted interventions to empower older informal workers in Ghana through secure technology adoption and digital skills building.

### Digital Literacy in Ghana

This digital age requires digital technologies and abilities to help enterprises (Kaeophanuek et al., 2018). Akpan et al. (2020) and Papadopoulos, (2020) highlighted the crucial role of technology in business growth in this digital age, emphasizing the need for all entrepreneurs to understand and utilize digital technologies effectively. Studies indicate a substantial disparity between the supply and demand of digital skills in the sub-Saharan Africa area, with a lower availability of skills compared to other markets, particularly at intermediate and advanced skill levels (Dovie et al., 2019). In Ghana, the digital divide affecting older adults is of particular importance and has been well-documented in the literature. As the country undergoes digital transformation across various sectors, the ability of older workers to effectively utilize digital tools and technologies has significant implications for their employability and productivity (Dovie et al., 2019; Frempong, 2012). L.E.K and the International Financial Corporation (IFC) (2019) emphasize the need to boost the availability of a digitally proficient workforce in Ghana to address future labour market demands.

Studies found that most older Ghanaian respondents were considered not digitally literate (Awotwi, 2015; Ussher, 2020). The studies identified several factors contributing to this digital divide, including lower educational attainment, rural residency, and advanced age. Radovanovic et al (2020) emphasized the need for targeted interventions to improve digital skills among the country’s growing ageing population, including those still in the workforce. Similarly, Ehimuan et al. (2024) found that lack of access to digital devices and affordable internet, as well as limited opportunities for digital skills training, were major impediments to technology adoption. The study called for policy actions and community-based programs to enhance digital literacy and access among older persons, including those seeking to maintain or re-enter the workforce. Earlier works by Ofori-Dwumfuo and Paatey (2011) also highlighted the cultural perceptions that discourage technology use among older Ghanaians. The study participants viewed digital skills as more relevant for younger generations, contributing to lower motivation and confidence in adopting new technologies - a mindset that can be particularly detrimental for older workers facing digital transformation in their industries. Recognizing these challenges, some organizations have implemented initiatives to improve digital literacy for older persons in Ghana, including efforts targeted at older workers. For instance, a study by International Telecommunication Union (2022) documented their efforts to deliver digital skills training workshops in rural communities, aiming to empower older adults to utilize digital tools and services. However, the scope and reach of such programs remain limited. Furthermore, the Ghanaian government has also acknowledged the need to address the digital divide for the ageing population, including older workers. The National Digital Literacy Program, led by the Ministry of Communications, has included older adults as a target beneficiary, though the implementation and impact of these efforts are still emerging (Abakah & Berko, 2023).

The existing literature therefore paints a concerning picture of the digital literacy challenges faced by older adults in Ghana. The studies have consistently found lower levels of digital literacy and technology adoption among this cohort compared to younger age groups. Moreover, digital literacy among older informal workers is a critical yet underexplored area of research. As digital technologies increasingly permeate all aspects of life, the ability to effectively use these technologies becomes essential for economic participation, social inclusion, and personal well-being. However, older informal workers often face unique challenges in acquiring digital literacy due to age-related factors, economic constraints, and the informal nature of their employment. Sustained, multi-stakeholder interventions are needed to bridge this divide and ensure equitable digital participation for Ghana’s ageing workforce, particularly among informal workers who major this population.

## Methodology

### Research Design

This study utilized a qualitative, descriptive single case study design. The case study approach was selected as it allows for an in-depth, holistic exploration of a phenomenon within its real-world context using multiple sources of data (Yin, 2014). As this research aimed to understand digital technology use among older informal workers in Kumasi, Ghana, the case study design enabled a localized, and contextualized analysis. Specifically, this study adopted an instrumental case study approach, focusing on a specific issue rather than the case itself (Grandy, 2010). The case facilitated an understanding of the broader issue of technology adoption among older informal workers. Kumasi’s central business district served as the bounded case providing opportunities to explore this issue. A descriptive approach was taken to provide a comprehensive summary of the technological behaviors, experiences, and perspectives described by the participants within the study’s context. Rich, descriptive data was collected through interviews to convey a detailed account of technology use from the participants’ point of view.

### Participants Selection and Eligibility Criteria

Purposive sampling was used to recruit participants who met specific inclusion and exclusion criteria relevant to the research objectives. The target population for this study was older adults over 50 years engaged in informal work in the Central Business District (CBD) of Kumasi. This age criterion aligns with the International Labor Organization’s (2020) conceptual definition of older workers in the labor market. Inclusion criteria were that participants had to be aged 50 years or older, currently engaged in informal economic activities, and be available for an in-person interview during the study period. Exclusion criteria were that participants could not be younger than 50 years old, work in the formal sector with regular wages and benefits, and be unavailable for an interview during the study period. A sample size of 27 participants was determined based on data saturation principles, which refers to the point when collecting additional data does not yield substantial new themes or information in qualitative research (Guest et al., 2020). Throughout the data collection and analysis process, researchers closely monitored the emergence of new themes and the repetition of key insights. By the 24th interview, the researchers began to observe a high degree of thematic consistency, with subsequent interviews yielding minimal new information. This pattern of data saturation, combined with insights from the pilot study, the homogenous characteristics of the older informal worker cohort, and precedent from similar studies in the literature, informed the researchers’ conclusion that a sample of 27 participants would provide sufficient data to robustly address the research questions at hand (Guest et al., 2020; Hennink et al., 2017).

### Data Collection Procedure

Primary data was collected through in-depth, semi-structured interviews conducted face-to-face with each participant. Semi-structured interviews allow for focused, conversational discussions guided by a set of pre-determined open-ended questions while also enabling impromptu follow-up questions as needed. The interviews lasted approximately 45 min to one hour and were conducted at the participants’ places of work, at times convenient for them. Before conducting the interviews, a pre-tested interview guide was done with a small pilot group of five (5) older informal workers in the Ejisu Municipality, another hub of informal economic activities in the Kumasi metropolitan area of the Ashanti Region. This pilot phase allowed the researchers to assess the clarity, flow, and appropriateness of the questions within the local context, as well as identify any gaps or areas that required further refinement. The researchers closely observed participant reactions, noted any questions that caused confusion or required additional probing, and timed the overall duration to ensure it aligned with the expected timeframe. After debriefing on the pilot interview observations, the researchers rephrased certain questions, added new probes, reordered the flow, and streamlined some redundant questions. Subsequently, the researchers conducted another round of pilot interviews with five (5) participants using the revised guide to ensure the changes adequately addressed the previous concerns. Through this iterative pilot testing and debriefing process, the research team was able to thoroughly validate the interview guide, optimizing it for eliciting rich, contextual data from the target population of older informal workers in the Kumasi metropolitan area of Ghana.

The interview guide used covered key topics aligned with the research objectives. Participants were first asked demographic questions about their age, education level, type of informal work, years of experience etc. The main interview questions then focused on types of digital technologies used for their income generation before and during the pandemic, how these technological devices specifically helped their life and business during the pandemic, challenges faced in terms of access, skills, trust etc. related to using digital devices, any formal or informal training received in using technology during the pandemic, and general attitudes, perceptions and recommendations related to technology use.

Participants were recruited directly at their places of work such as markets, roadside vending areas, lorry stations, and artisanal workshops located throughout the CBD. The researchers approached potential participants who appeared to meet the inclusion criteria at these sites and invited them to take part in the study. Interviews were conducted face-to-face at the work sites during times convenient for participants, enabling observers to directly see the technologies used within the contextual realities of the participants’ occupations. However, some interviews were conducted in the homes of participants to accommodate their preferences and schedules. Prior to each interview, verbal informed consent was obtained after explaining the study’s purpose, voluntary nature, potential risks and benefits, and privacy protections. Interviews were audio recorded with permission, along with detailed handwritten notes. Data collection took place over 6 weeks from January to March 2023, intending to interview 4–5 older informal workers per week across various CBD locations based on feasibility and availability. All data including field notes, recordings, transcripts, and consent forms were de-identified and securely stored to maintain participant confidentiality.

### Data Analysis Procedure

The interview transcripts were analyzed using thematic analysis, a qualitative method that involves identifying, analyzing, and reporting patterns or themes within data (Clarke & Braun, 2017). This enabled a rich, detailed, and nuanced account of the dataset. Analysis was facilitated using NVivo 12 qualitative data analysis software, where tools like word frequency, text search, and matrix coding queries facilitated deeper analysis. The transcripts were thoroughly read to gain familiarity, and then systematically coded by segmenting the text into categories representing themes and patterns. Open coding was first used to analyze each line and extract initial concepts from the raw data inductively. Axial coding then organized these concepts into broader categories based on relationships between the open codes. Finally, selective coding integrated and refined the categories to determine overarching themes and sub-themes. A hybrid approach of inductive and deductive coding was used, allowing themes to emerge directly from the data as well as informed by the study objectives and interview guide. The lead researcher conducted the coding and theme development, with two other researchers independently coding a subset of transcripts. Coding comparisons ensured consistency and rigor. The key themes were analyzed, interpreted, and discussed in relation to the research questions and literature. Descriptive accounts conveyed the essence of the participants’ experiences in their own words under each theme. The direct quotes in the study illustrated themes in the presentation of findings.

### Ethical Considerations

The researchers conducted this study in accordance with robust ethical research principles, as approved by the Humanities and Social Sciences Research Ethics Committee, as part of a larger study on ageism and health and wellbeing of older informal workers in Ghana (HuSSREC/AP/64/VOL1). Informed consent was meticulously obtained through a printed form that clearly explained the study’s purpose, procedures, potential risks and benefits, privacy protections, and the voluntary nature of participation. Participants provided their signatures or thumbprints to consent to the study. To maintain participant privacy and confidentiality, findings were reported without identifying individual participants. The researchers also ensured that interviews were scheduled at convenient times to minimize work disruptions for the participants.

## Findings

### Demographic Characteristics

The study included a total of 27 participants, comprising 16 females and 11 males, aged between 50 and 62 years. Regarding education, nearly half of the participants had no formal education, while a substantial portion had completed Senior High School. Marital status varied among participants, with the majority being married. In terms of employment, participants were involved in various informal occupations, with dressmaking, street vending, and trading being the most common. Detailed demographic characteristics of the participants are provided in Table 1.

**Table 1:** Characteristics of study participants

Pseudonyms	Gender	Age	Educational level	Marital status	Employment
1st	Female	50	Senior high School	Married	Dressmaking
2nd	Male	60	Senior High School	Married	Dressmaking
3rd	Female	59	No education	Widowed	Street vendor
4th	Female	53	Junior High School	Married	Food vendor
5th	Female	51	No education	Widowed	Street vendor
6th	Male	62	Senior High School	Married	Driver
7th	Male	54	No education	Separated	Driver
8th	Female	50	Junior High School	Married	Street vendor
9th	Male	55	No education	Widowed	Waste/Scrub collector
10th	Female	52	Junior High School	Single	Trading
11th	Male	57	Senior High School	Separated	Shoemaker
12th	Male	55	Junior High School	Single	Repairer (electronics)
13th	Female	50	No education	Separated	Food vendor
14th	Male	59	No education	Single	Driver
15th	Female	61	Tertiary	Married	Trader
16th	Female	60	No education	Single	Food vendor
17th	Male	50	Junior High School	Single	Street vendor
18th	Female	54	No education	Married	Trader
19th	Female	60	Junior High School	Separated	Trading
20th	Female	58	Junior High School	Married	Dressmaking
21st	Male	51	No education	Married	Plumbing
22nd	Female	50	Vocational School	Married	Trading
23rd	Male	50	Junior High School	Married	Trading
24th	Female	50	Junior High School	Married	Trading
25th	Female	51	Tertiary	Married	Trading
26th	Female	50	Junior High School	Married	Dressmaking
27th	Male	60	Senior High School	Married	Trading

*Source: Authors’ construct*

### Main Findings

The analysis revealed that older informal workers expressed both positive and negative ways technological devices impacted their businesses during the pandemic era. Specifically, four ways revealing diverse potentials and challenges of technological devices on older informal workers are as follows: an inclination to mobile phone usage during the pandemic; navigating pandemic barriers through technology to improve businesses; technology-induced barriers hampering businesses during the pandemic; absence of formal digital skills training and reliance on informal assistance. The themes that emerged have been discussed as follows:

#### Inclination to Mobile Phone Usage during the Pandemic

The findings provided insights into the type of technological device commonly used by informal older workers. Participants highlighted that they relied heavily on their mobile phones as a critical technological tool to sustain their businesses during the COVID-19 pandemic. Participants observed that they preferred mostly to use mobile phones than any other technological devices because it is easily accessible and reliable:

*“I always use my phone…. I use it everyday because mobile phones are everywhere. Many people including those of us who are not educated can have access to phones. It is easy to buy and also makes work easier for me.” Participant 5*.

*“When the pandemic hit*,* I had to find a way to stay connected with my clients without being able to meet them in person. My mobile phone became my lifeline - I could call them*,* text*,* and even send pictures of my work.” – Participant 18*.

Some participants, believed that while making an effort to use other technological devices, mobile phone usage was easier to operate as compared to other technological devices, especially during the pandemic. For others, the decision to opt for this device was due to its accessibility and cost friendliness. Participants had this to say:

*“During the COVID-19 pandemic*,* I used a mobile phone every day. Though I was thinking of getting a laptop and a tablet*,* my personal phone was what I used always because it was much easier to operate.” Participant 15*.

*“Using the phone in doing my business is not expensive. I have two phones*,* one is a “yam phone” [non-smartphone]*,* and another that supports WhatsApp. ”During the pandemic*,* all I only needed was to buy MTN credits to call my customers and to also post some of my clothes on WhatsApp. I think using the phone to do this is much easier.” Participant 20*.

Other participants’ also emphasized reliance on their mobile phones on a near-constant, all-day basis during the COVID-19 lockdowns. The frequency of use portrayed how integral the devices was in sustaining their informal businesses and livelihoods when in-person activities were restricted.

*“During the entire COVID-19 lockdown period*,* I was on my mobile phone constantly - using it to take photos of my products*,* posting them on WhatsApp and Facebook*,* chatting with customers*,* and coordinating deliveries. It was the only way I could keep my small trading business going.” - Participant 11*.

*“From morning till night*,* my mobile phone was glued to my hand as I worked at home. I was making calls and sending voice messages to coordinate with clients*,* and even video calling them to show the progress on their shoes. It was the only way I could continue my shoe making when my shop was closed during the pandemic lockdowns.” – Participant 26*.

*“As a driver*,* my mobile phone was essential for accepting ride requests*,* communicating with passengers*,* and even taking digital payments. I was on that thing all the time during COVID just to keep my business afloat.” – Participant 7*.

#### Navigating Pandemic Barriers through Technology Usage to Improve Businesses

The study participants discussed how they leveraged technology to engage in their business operations during the COVID-19 pandemic restrictions. The findings suggest that mobile phones which were the only technological devices accessible to participants, provided significant benefits for them during pandemic. First, mobile devices were used by the participants to advertise their products and services through social media platforms, such as WhatsApp and Facebook. This allowed them to reach out to customers and maintain business connections despite the pandemic-related mobility constraints and social distancing measures. The ability to connect with customers digitally proved crucial for many informal workers to sustain their livelihoods when face-to-face interactions were limited.

*“During the lockdown*,* I started using my mobile phone to advertise my dresses on WhatsApp. It was amazing to see how many customers reached out to me through the app*,* even when they couldn’t come to my shop.” – Dressmaker”. Participant 22*.

The study participants further discussed how they used mobile money services to transfer and receive payments from their customers, which was beneficial in reducing workload during the pandemic and has now sustained their businesses. Participants, particularly older men reported that by registering their mobile phones with mobile money services, they were able to engage in digital financial transactions with their clients without the need for in-person meetings. This enabled them to continue conducting business smoothly, even as physical interactions were limited due to the pandemic restrictions.

As one participant noted:

*“Using my phone for e-payments has really helped. I have customers in and outside Kumasi. I don’t have to travel to collect money or make payments anymore. It saved me a lot of time and effort*,* especially when movement was restricted.” Participant 10*.

The ability to facilitate digital payments through their mobile devices allowed participants to streamline their financial transactions and minimize the logistical challenges posed by COVID-19. This flexibility in managing payments contributed to the participants’ capacity to maintain business continuity during this disruptive period. Moreover, the participants also highlighted how the use of mobile phones has helped protect them from theft and robbery. They expressed that the ability to conduct financial transactions digitally, rather than relying on physical cash, has been a significant benefit in enhancing their safety.

*“Oh*,* it has helped me to be alert about the mobile money fraudsters. Also*,* I do not need to keep physical cash. All my money exists in the form of e-cash on my phone*,* so those thieves do not attack me any longer.” - Participant 16*.

The shift away from carrying large amounts of physical cash has provided them with a greater sense of security in their day-to-day business operations. Additionally, male participants expressed that they have become more vigilant about potential mobile money scams and fraud, further enhancing their ability to safeguard their financial assets. The use of digital payment methods has, therefore, not only improved their business efficiency but also contributed to their personal safety and risk mitigation.

While some participants were able to leverage technology to support the survival of their business operations during the pandemic period, many participants still experienced the collapse of their businesses despite the availability of mobile phones. They complained that since their businesses mostly required face to face interaction with clients, the presence of technological device was not beneficial to their businesses and led to its collapsed. One of the participants said:

*“During the pandemic I was not working*,* and I could not use my phone to do my business either. The nature of my work requires face to face contact and people do not always come as before so I could not use my phone in transacting business.” Participant 21*.

Another participant who worked as scrub collector shared a similar concern by saying that:

*“My business requires no technological device. We work by collecting scrubs from the streets. However*,* the lockdowns prevented us from going to work and I forfeited my daily income. This is how my business and income was affected.” Participant 9*.

Another participant also added her voice by saying that without access to technology, she had a very hard time adapting their businesses during the pandemic. She could not effectively advertise, connect with customers, or manage operations remotely.

*“The pandemic just destroyed my business. The lockdown shut everything down*,* including my street stall*,* for a long time. But then people started needing masks*,* so I tried making some*,* even though I am not a professional seamstress. It helped a bit*,* but overall*,* it was not very successful. Without any way to use technology to sell or promote my products*,* I really struggled to keep my business going during the COVID crisis.” Participant 19*.

Thus, the lack of digital tools made it much more challenging to keep participants’ businesses going through the disruption.

#### Technology Induced Barriers Hampering Businesses

Participants were also asked to share the challenges they faced in using technology for their businesses during the pandemic. The narratives suggested that several factors posed major barriers and made participants particularly vulnerable, impeding the sustainability of their enterprises. Key issues included the prevalence of mobile phone fraudsters, data corruption, scamming, and poor internet connectivity on their mobile devices.

Participants, especially the older women, felt that mobile money transfers exposed them to fraudsters and scammers, which significantly hindered their business operations. They reported losing substantial sums of money due to these mobile money scams, which severely undermined their ability to maintain their businesses during the pandemic disruption. The lack of reliable technology and the threat of financial fraud created an environment of heightened risk and insecurity for the older informal workers as they tried to adapt their practices.

*“Fraud is an issue*,* one day I was here*,* and I received a call from someone who mentioned my name that my sister who is abroad has sent me some items to be delivered to me. So later I realized it was one of the fraudsters. Not knowing they were a team. Another also called me the next day and said that he had sent money to me on mobile money. Unfortunately*,* I became a victim since I gave the number to a sister who transferred 11 million to these fraudsters. So*,* in a way the phone has helped but it is a necessary evil.” Participant 3*.

Some participants also shared this view but added they have been perpetual victims of thieves who always steal the mobile phone used to engage in their business. They complained that thieves stole their phones to lure their customers to fall prey to their nebulous acts and siphon money from them. One of them explained that:

*“Ooh hmm this is the fourth time thieves have stolen my phone. They always steal the phone, to lure my customers and steal money from them. I have received several reports from my customers in this pandemic time who have been duped by thieves using my own phone*,* this is my only problem.” Participant 4*.

Other participants complained about poor internet connectivity on their mobile phones. They complained that unreliable and inadequate internet connectivity posed a significant barrier for their livelihood. Additionally, some participants expressed frustration over accidentally deleting business records due to a lack of digital literacy and insufficient knowledge in properly using their phones. One of the participants had this to say:

*The internet connection is bad here. Sometimes, it hard for me to make a simple call or use the internet for my business. You can sometimes lose important information too on your phone. Some of the smartphones require in-depth knowledge to operate and because of this*,* I accidentally delete client’s information which affects me” Participant 23*.

Despite the challenges they faced with technology, most participants acknowledged that using it was essential for the survival and sustainability of their businesses during the pandemic. Even though they encountered issues like fraud and connectivity problems, they were reluctant to avoid using technology, as it was their only means of keeping their enterprises afloat. One of them had this to say:

*“Sometimes*,* I decide not to use the phone in my work because of fraudsters but I later realized that I have to use it if I want to succeed in this job especially during the pandemic.” Participant 5*.

However, few of the participants, particularly older men expressed how cautious and tactical they have become in dealing with the fraudsters they encounter. For instance, one of them said this:

*“I have issues with the fraudsters*,* but you need to be careful. Sometimes fraudsters call me but since I am smart*,* I always redirect them to the MTN office to take their money if indeed what they are saying is true.” Participant 27*.

#### Absence of Formal Digital Skills Training and Reliance on Informal Assistance

Most participants were of the opinion that they had not attained any formal digital skill training to improve their businesses. This instance was mainly attributed to, reluctance on their side to learn and apathy on the part of government and mobile network operators to teach them how to upgrade their digital literacy and skills in ensuring the sustainability of the businesses. The views are summarized below:

*“Hmmm*,* up until now I do not know how to properly operate this smartphone*,* especially when it comes to using mobile money services. I have to rely on someone else here who assists me*,* as I have not made the effort to learn these digital skills myself. I can check my account balance*,* but when someone sends me money*,* I am unable to transfer it on my own. This leaves me vulnerable to potentially being taken advantage of.” - Participant 8*.

*“No*,* I do not receive any assistance and training. Everything depends on you. I think the government and the network companies are not ready to teach us how to deal with these fraudsters. You just need to be careful and know their tricks that’s all.” Participant 6*.

However, the lack of formal training opportunities meant that participants had to rely on their immediate family members to acquire the knowledge and skills needed to effectively use technology for their business activities. This informal, familial support was essential for enabling them to continue operating their enterprises during the pandemic.

*“I have received training from my children and grandchildren on how to use my phone and to receive and send mobile money. Because of that when someone sends me money now*,* I see it, and I am able to follow the prompts to withdraw the money. Also*,* I can call some of my customers by using the phone.”. Participant 11*.

*“Since my husband share such stories on fraudsters with me*,* I am always smart and careful about the fraudsters. He has been teaching me how to use the phone to detect such cases. He is more educated than me*,* so I rely on him for education and training.” Participant 5*.

Furthermore, a few participants indicated they acquired help from some of the mobile money vendors who assisted them regularly in money transactions. One mentioned that:

*“..... regarding training to use a phone, I have not acquired any. There is a mobile money vendor here who always assists me to withdraw my money.” Participant 21*.

## Discussion

The findings in the study reveal how deeply entrenched mobile digital devices have become among older informal workers in recent years, particularly during the COVID-19 pandemic. Their ubiquity, simplicity of use, and adaptability to diverse functions from communication to marketing have rendered phones an indispensable tool enabling digital and work engagement among older informal workers. This highlights the importance of ensuring continued mobile phone accessibility and affordability as a priority for supporting this cohort. It also suggests that digital literacy initiatives have a familiar and relevant starting point to build upon by leveraging mobile phones’ familiarity (Charness & Boot, 2009; Piper et al., 2017). While the absence of data consumption rate is a limitation, the consistent reports of frequent, regular technology engagement across participants still provides valuable insights. It demonstrates that this population, often assumed to have low digital literacy, was in fact actively leveraging various technologies to navigate the challenges of the pandemic. With guidance, older informal workers could further capitalize on mobile technology for empowerment, especially as social media opens new avenues for business promotion and sales (Saleh, 2021; Bocconcelli et al., 2017). However, the findings also surface a substantial gap in digital skills and formal training among older informal workers. Many lack the knowledge to fully capitalize on mobile phones, while limited literacy also leaves them vulnerable to over-reliance on others for basic functions as well as increased fraud risk. This underscores the need for authorities and telecommunication companies to prioritize digital literacy programs focused on basic mobile phone use and fraud prevention knowledge (Hecker et al., 2021; Ince, 2022). While willingness to learn exists among older workers, structured training opportunities remain inadequate currently in informal work settings. A collaborative approach engaging government agencies, private sector telecoms, non-profits agencies will be essential to provide comprehensive and accessible digital skills building (Chetty, 2023; Rakhmawan, 2022).

Additionally, the findings highlight the reliance of older informal workers on intergenerational and community collaboration to adapt to technology during the pandemic. The study emphasizes the importance of knowledge-sharing from the younger family members, suggesting that training initiatives should involve youth and digitally savvy relatives to help build mobile proficiency among older workers (Freeman et al., 2020; Reis et al., 2021) and facilitate technology adoption more effectively than formal interventions alone (Lee & Kim, 2019). Moreover, the guidance from spouses and mobile vendors, as observed in the study, underscores the value of informal community assistance for sustained technological engagement by less tech-savvy individuals (Elers et al., 2018). These systems can be designed to build on their existing knowledge and gradually introduce more advanced digital skills in a supportive environment. This is because the low levels of education and digital literacy among older informal workers necessitate more personalized and accessible learning strategies (Dovie, 2018; Neumeyer et al., 2020). Thus, while structured training programs is crucial, grassroots approaches tapping into these interconnected support networks will likely prove vital for achieving sustainable adoption and use of mobile phones and other technologies. Also, creating awareness about the availability and utility of online learning tools through targeted outreach and support can help older informal workers make better use of these resources (Jin et al., 2019).

Essentially, the COVID-19 pandemic underscored vital considerations regarding the role of mobile phones and digital literacy for older informal workers. This technology emerged as an indispensable tool enabling many participants to maintain business continuity amid mobility restrictions. Many older informal workers were able to leverage mobile phones and apps like WhatsApp to advertise their products, conduct e-payments, and reduce the need for in-person transactions and travel. This enabled them to continue generating income and serving customers, despite lockdowns and mobility restrictions. This highlights the importance of mobile connectivity and digital financial services in supporting informal economic activities, especially to cope with their livelihood during unprecedented crisis such as the pandemic (Lai & Widmar, 2021; Kimuli et al., 2021). It demonstrates how technology can enhance the resilience and adaptability of the informal sector, which is a critical component of many developing economies (Dayagbil et al., 2021; Sixsmith et al., 2022). Consistent with previous studies, mobile phones allowed adaptation to an unprecedented crisis by facilitating remote engagement and transactions (Elimelech et al., 2022; Datta & Nwankpa, 2021).

However, the findings reveal a dichotomy of outcomes. While many leveraged mobile phones effectively, others experienced severe business declines during the pandemic despite technology’s prevalence. Several factors contributed to this disparity in adaptation. First, mobile phones appeared to benefit workers whose businesses could operate remotely through digital engagement and transactions. Yet, participants reliant on in-person services seemed to struggle with digitization. Their persistent dependence on face-to-face interactions inhibited technology from alleviating pandemic pressures. Second, digital literacy likely influenced outcomes. Workers lacking skills to capitalize on mobile phones’ potential were less equipped to adapt their operations. Those with greater digital proficiency seemed more agile in leveraging technology under duress. Ultimately, the pandemic underscored mobile phones’ significance while surfacing important nuances during the pandemic (Elimelech et al., 2022; Sixsmith, 2020). It is also evident from the study that while expanding mobile phone access and digital skills training, remaining cognizant of limitations for workers reliant on in-person interactions will be prudent (Datta & Nwankpa, 2021). Consequently, as older informal workers navigate growing technological demands, a nuanced understanding of this cohort’s diversity is key for effective policies and support.

The findings also reveal that older informal workers face a significant challenge of mobile phone fraud, theft and data corruption as they navigate the technological landscape. This placed them at greater risk of financial losses and undermined their already precarious livelihoods. Although older informal workers adopted a range of risk mitigation measures, such as relying on trusted social networks, diversifying income sources, and using mobile money platforms, these strategies are not always fully effective, and many older informal workers remain highly vulnerable to fraud and its devastating consequences. Despite persistent threats, older informal workers remained committed to leveraging technology for business survival. The vulnerabilities to deception and criminal exploitation underscore the need for safeguarding measures integrated into training programs, to help identify and avoid scams (Efijemue et al., 2023). It is also interesting to note that the continued reliance on mobile phones despite risks indicates that policies should focus on mitigating threats rather than avoiding technology altogether (Charness & Boot, 2009; Choi et al., 2020). Also, the adaptability shown in confronting threats points to an opportunity for peer sharing of protective strategies and fraud prevention tips. Leveraging experienced individuals as resources could help collectively strengthen resilience.

Furthermore, while this study treated the cohort of older informal workers as a cohesive group, it is crucial to recognize the differences in digital adoption and capabilities across various categories of informal work. Each category of work presents unique opportunities and challenges regarding the use of digital technologies. These variations highlight the intersectionality between the type of informal work and the propensity to adopt and utilize technology. For instance, while some workers leveraged technology to enhance their business visibility and efficiency, others were restricted by the nature of their work and the limited functionalities of their devices. This points to a significant need for targeted training and the provision of more advanced and accessible technologies to support older informal workers across different sectors. In addition, while the generational gap in digital literacy is established in extant literature, findings of this study provide substantial evidence of the intersectionality of factors such as gender and socioeconomic status, in shaping digital literacy among older workers, particularly those working in the informal sector. Whiles studies highlight the significant role of gender and socio-economic status in digital literacy among older adults (Tirado-Morueta et al., 2018; Neves et al., 2013), they have shown mixed results, particularly among older informal workers. Understanding these distinctions is crucial for developing policies that cater to the diverse needs of informal worker groups. Targeted support can empower older informal workers to stay digitally included, economically resilient, and employable in the post-pandemic era.

## Conclusion

This study provides valuable insights into the intersection of digital technology and livelihoods among older informal workers in Ghana during the COVID-19 pandemic. It highlights the growing integration of mobile phones into income-generating activities of older informal workers, underscoring their significance in maintaining business continuity, especially during the COVID-19 pandemic. Despite their potential, significant barriers to sustainable adoption persist, including limited digital literacy, lack of structured training, connectivity issues, and the threat of fraud and other cyber risks. The findings emphasize the need for targeted policies and an ecosystem approach to unlock the potential of digital tools while mitigating associated risks. Expanding mobile-centric training programs tailored to diverse literacy levels, coupled with robust social support systems, can strengthen digital adoption among older informal workers. The study emphasizes that lifelong learning initiatives are vital for extending working lives in the post-pandemic era. Therefore, integrating continuous learning opportunities with social protection measures will ensure older informal workers adapt to rapid technological changes. These initiatives should focus on not only digital literacy but also broader educational needs to enhance overall well-being and economic resilience. Ultimately, the resilience of Ghana’s ageing workforce demonstrates their potential to adapt with the right support. Lessons from this cohort can inform policies and interventions across the Global South, addressing similar dynamics and fostering greater empowerment and prosperity in the informal sector. With appropriately nuanced interventions, digital technologies can provide pathways to enhanced economic resilience and inclusion for older informal workers in this post-pandemic era.

### Limitations of the Study

This study presents several limitations that should be acknowledged to provide a comprehensive understanding of its scope and applicability. First, the geographic focus on the Central Business District of Kumasi and the reliance on self-reported data from semi-structured interviews may limit the generalizability of the findings to other regions or countries with different economic, social, and technological contexts. Future research could broaden the geographic scope in tandem with triangulating interview data with other data sources, such as quantitative data, observational studies or digital usage analytics, to enhance generalizability. Second, a notable limitation of this study is the absence of insights from key stakeholders beyond the older informal workers themselves. The paper did not incorporate perspectives from policymakers, digital literacy training providers, or other relevant stakeholders who could offer valuable insights on the feasibility and implementation of potential solutions. Engaging with these stakeholders could have provided a more comprehensive understanding of the challenges and opportunities for strengthening digital literacy among older informal workers. Despite these limitations, the study’s contributions to the understanding of digital technology integration among older informal workers are significant. It offers valuable insights and lays the groundwork for future research and policy development aimed at enhancing digital inclusion and economic resilience in the informal sector.

## Data Availability

The data used to support the findings of this study are not publicly available due to privacy or confidentiality restrictions. However, the data that support the findings of this study are available on request from the corresponding author, [OAS].

## References

[CR1] Abakah, E., & Berko, K. K. (2023). Marginalisation of older adults in the digitalisation drive in ghana: Exploring the potential of adult learning and education. *Promoting the Socio-Economic Wellbeing of Marginalized individuals through Adult Education* (pp. 176–201). IGI Global. 10.4018/978-1-6684-6625-4.ch010

[CR2] Akpan, I. J. (2020). Scientometric evaluation and visual analytics of scientific literature production on entrepreneurship, small business ventures, and innovation. *Journal of Small Business & Entrepreneurship*, 1–29. 10.1080/08276331.2020.1786229

[CR3] Anakpo, G., & Mishi, S. (2021). Business response to COVID-19 impact: Effectiveness analysis in South Africa. The southern African. *Journal of Entrepreneurship and Small Business Management*, *13*(1), 7. 10.4102/sajesbm.v13i1.397

[CR43] L.E.K and International Financial Corporation (2019). *Digital Skills in Sub-Saharan Africa Spotlight on Ghana* https://www.ifc.org/wps/wcm/connect/ed6362b3-aa34-42ac-ae9f-c739904951b1/ Digital + Skills_Final_WEB_5-7-19.pdf?MOD = AJPERES (11/11/2019).

[CR4] Asongu, S. A. (2018). Conditional determinants of mobile phones penetration and mobile banking in Sub-saharan Africa. *Journal of the Knowledge Economy*, *9*(1), 81–135. 10.1007/s13132-015-0322-z

[CR260] Awotwi, J. E. (2015). *Challenging the digital divide in a developing country: Ghana case study*. Digital Divides, 47.

[CR5] Bartik, A. W., Bertrand, M., Cullen, Z., Glaeser, E. L., Luca, M., & Stanton, C. (2020). The impact of COVID-19 on small business outcomes and expectations. *Proceedings of the National Academy of Sciences*, *117*(30), 17656–17666. 10.1073/pnas.2006991117 PMC7395529 32651281

[CR6] Bocconcelli, R., Cioppi, M., & Pagano, A. (2017). Social media as a resource in SMEs’ sales process. *Journal of Business & Industrial Marketing*, *32*(5), 693–709. 10.1108/JBIM-11-2014-0244

[CR7] Bonnet, F., Vanek, J., & Chen, M. (2019). Women and men in the informal economy: A statistical brief. *International Labour Office Geneva*, *20*, 22.

[CR8] Budnitz, H., & Tranos, E. (2022). Working from home and digital divides: Resilience during the pandemic. *Annals of the American Association of Geographers*, *112*(4), 893–913.

[CR9] Cebola, M. M. J., dos Santos, N. R., & Dionísio, A. (2023). Worker-related ageism: A systematic review of empirical research. *Ageing & Society*, *43*(8), 1882–1914.

[CR10] Charness, N., & Boot, W. R. (2009). Aging and information technology use: Potential and barriers. *Current Directions in Psychological Science*, *18*(5), 253–258.

[CR11] Chetty, K. (2023). AI literacy for an ageing workforce: Leveraging the experience of older workers. *OBM Geriatrics*, *7*(3), 1–17.

[CR12] Choi, J., Dutz, M. A., & Usman, Z. (Eds.). (2020). *The future of work in Africa: Harnessing the potential of digital technologies for all*. World Bank.

[CR13] Clarke, V., & Braun, V. (2017). Thematic analysis. *The Journal of Positive Psychology*, *12*(3), 297–298.

[CR14] Datta, P., & Nwankpa, J. K. (2021). Digital transformation and the COVID-19 crisis continuity planning. *Journal of Information Technology Teaching Cases*, *11*(2), 81–89.

[CR15] Dayagbil, F. T., Palompon, D. R., Garcia, L. L., & Olvido, M. M. J. (2021, July). *Teaching and learning continuity amid and beyond the pandemic*. *In Frontiers in Education* (Vol. 6, p. 678692). Frontiers Media SA.

[CR16] Dovie, D. A. (2018). Financial literacy in an African society: An essential tool for retirement planning. *Contemporary Journal of African Studies*, *5*(2), 26–59.

[CR17] Dovie, D. A., Dzorgbo, D. B. S., Mate-Kole, C. C., Mensah, H. N., Agbe, A. F., Attiogbe, A., & Dzokoto, G. (2019). Generational perspective of digital literacy among ghanaians in the 21st century: Wither now? *Media Studies*, *10*(20), 127–152.

[CR18] Efijemue, O., Obunadike, C., Taiwo, E., Kizor, S., Olisah, S., Odooh, C., & Ejimofor, I. (2023). Cybersecurity strategies for safeguarding customers data and preventing financial fraud in the United States financial sectors. *International Journal of Soft Computing*, *14*(3), 10–5121.

[CR19] Ehimuan, B., Anyanwu, A., Olorunsogo, T., Akindote, O. J., Abrahams, T. O., & Reis, O. (2024). Digital inclusion initiatives: Bridging the connectivity gap in Africa and the USA–A review. *International Journal of Science and Research Archive*, *11*(1), 488–501.

[CR20] Elers, P., Hunter, I., Whiddett, D., Lockhart, C., Guesgen, H., & Singh, A. (2018). User requirements for technology to assist aging in place: Qualitative study of older people and their informal support networks. *JMIR mHealth and uHealth*, 6(6), e10741. 10.2196/10741 PMC6010833 29875083

[CR21] Elimelech, O. C., Ferrante, S., Josman, N., Meyer, S., Lunardini, F., Gómez-Raja, J., & Rosenblum, S. (2022). Technology use characteristics among older adults during the COVID-19 pandemic: A cross-cultural survey. *Technology in Society*, *71*, 102080. 35991080 10.1016/j.techsoc.2022.102080 PMC9376146

[CR22] Erlanitasari, Y., Rahmanto, A., & Wijaya, M. (2020). Digital economic literacy micro, small and medium enterprises (SMES) go online. *Informasi*, *49*(2), 145–156.

[CR23] Freeman, S., Marston, H. R., Olynick, J., Musselwhite, C., Kulczycki, C., Genoe, R., & Xiong, B. (2020). Intergenerational effects on the impacts of technology use in later life: Insights from an international, multi-site study. *International Journal of Environmental Research and Public Health*, *17*(16), 5711. 32784651 10.3390/ijerph17165711 PMC7459619

[CR24] Frempong, G. (2012). *Understanding What is Happening in ICT in Ghana: A Supply- and Demand-side Analysis of the ICT Sector*. Evidence for ICT Policy Action Policy Paper 4, 2012.

[CR25] Grandy, G. (2010). Instrumental case study. *Encyclopedia of Case Study Research*, *1*, 473–475.

[CR26] Guest, G., Namey, E., & Chen, M. (2020). A simple method to assess and report thematic saturation in qualitative research. *PloS One*, 15(5), e0232076. 10.1371/journal.pone.0232076 PMC7200005 32369511

[CR27] Hecker, I., & Briggs, A. (2021). *Overlooked and underconnected: Exploring disparities in digital skill levels by race among older Youth in the US*. Urban Institute.

[CR28] Hecker, I., & Loprest, P. (2019). Foundational Digital Skills for Career Progress. *Urban institute*.

[CR29] Hecker, I., Spaulding, S., & Kuehn, D. (2021). Digital skills and older workers. *Urban Institute*, *2021*, 1–24.

[CR30] Hennink, M. M., Kaiser, B. N., & Marconi, V. C. (2017). Code saturation versus meaning saturation: How many interviews are enough? *Qualitative Health Research*, *27*(4), 591–608. 27670770 10.1177/1049732316665344 PMC9359070

[CR31] Hunsaker, A., & Hargittai, E. (2018). A review of internet use among older adults. *New Media & Ssociety*, *20*(10), 3937–3954.

[CR32] ILO. (2015). *World employment and social outlook: Trends 2015*. International Labour Organization.

[CR33] ILO. (2018). *Women and men in the informal economy: A statistical picture*. International Labour Office.

[CR35] ILO. (2020). *ILO Monitor: COVID-19 and the World of Work*. International Labor Organization.

[CR34] ILO (2020a). Promoting Employment and Decent Work in a Changing Landscape: 2020 General Survey. ILC109/III(B). Geneva.

[CR36] Ince, F. (2022). *Digital Literacy Training: Opportunities and Challenges*. Handbook of Research on the Role of Libraries, Archives, and Museums in Achieving Civic Engagement and Social Justice in Smart Cities, 185–199.

[CR37] International Telecommunication Union (ITU) (2022). Ghana’s underserved rural communities get a digital skills training boost. ITU News. Retrieved from https://www.itu.int/hub/2022/11/ghana-digital-skills-training-dtci/

[CR38] Jin, B., Kim, J., & Baumgartner, L. M. (2019). Informal learning of older adults in using mobile devices: A review of the literature. *Adult Education Quarterly*, *69*(2), 120–141.

[CR39] Kaeophanuek, S., Na-Songkhla, J., & Nilsook, P. (2018). How to enhance digital literacy skills among information sciences students. *International Journal of Information and Education Technology*, *8*(4), 292–297.

[CR40] Kimuli, S. N. L., Sendawula, K., & Nagujja, S. (2021). Digital technologies in micro and small enterprise: Evidence from Uganda’s informal sector during the COVID-19 pandemic. *World Journal of Science Technology and Sustainable Development*, *18*(2), 93–108.

[CR41] Lai, J., & Widmar, N. O. (2021). Revisiting the digital divide in the COVID-19 era. *Applied Economic Perspectives and Policy*, *43*(1), 458–464. 33230409 10.1002/aepp.13104 PMC7675734

[CR42] Lee, O. E. K., & Kim, D. H. (2019). Bridging the digital divide for older adults via intergenerational mentor-up. *Research on Social Work Practice*, *29*(7), 786–795.

[CR44] Meliou, E., Mallett, O., & Rosenberg, S. (2019). Being a self-employed older woman: From discrimination to activism. *Work Employment and Society*, *33*(3), 529–538.

[CR46] Neumeyer, X., Santos, S. C., & Morris, M. H. (2020). Overcoming barriers to technology adoption when fostering entrepreneurship among the poor: The role of technology and digital literacy. *IEEE Transactions on Engineering Management*, *68*(6), 1605–1618.

[CR45] Neves, B. B., Amaro, F., & Fonseca, J. R. (2013). Coming of (old) age in the digital age: ICT usage and non-usage among older adults. *Sociological Research Online*, *18*(2), 22–35.

[CR47] Ofori-Dwumfuo, G. O., & Paatey, E. (2011). The design of an electronic voting system. *Research Journal of Information Technology*, *3*(2), 91–98.

[CR48] Oteng, S. A., Manful, E., & Akuoko, K. O. (2022). Retiring in the informal economy: Implications for social policy intervention for ageing workers in Ghana. *Ageing International*, *47*(3), 415–432.

[CR49] Oteng, S. A., Amoah, P. A., & Huang, G. (2024). Deconstructing ageism among older informal workers: A systematic review. *International Journal of Sociology and Social Policy*.

[CR50] Papadopoulos, T., Baltas, K. N., & Balta, M. E. (2020). The use of digital technologies by small and medium enterprises during COVID-19: Implications for theory and practice. *International Journal of Information Management*, *55*, 102192. 32836646 10.1016/j.ijinfomgt.2020.102192 PMC7327446

[CR51] Petrova, K. (2019). Globalization and the informal economy in developing countries. *Globalization and development: Economic and socio-cultural perspectives from emerging markets*, 49–73.

[CR52] Phillipson, C. (2019). Fuller or extended working lives? Critical perspectives on changing transitions from work to retirement. *Ageing & Society*, *39*(3), 629–650.

[CR53] Piper, A. M., Brewer, R., & Cornejo, R. (2017). Technology learning and use among older adults with late-life vision impairments. *Universal Access in the Information Society*, *16*(3), 699–711.

[CR263] Radovanovic, D., Holst, C., Belur, S. B., Srivastava, R., Houngbonon, G. V., Le Quentrec, E., Miliza, J., Winkler, A. S., & Noll, J. (2020). Digital literacy key performance indicators for sustainable development. *Social Inclusion, 8*(2), 151–167.

[CR54] Rakhmawan, S. A. (2022). Digital Transformation of Informal Workers in the New Normal era: Can it be the solution we are searching for? *East Java Economic Journal*, *6*(2), 182–207.

[CR55] Ratten, V. (2020). Coronavirus (covid-19) and entrepreneurship: Changing life and work landscape. *Journal of Small Business & Entrepreneurship*, *32*(5), 503–516.

[CR56] Reis, L., Mercer, K., & Boger, J. (2021). Technologies for fostering intergenerational connectivity and relationships: Scoping review and emergent concepts. *Technology in Society*, *64*, 101494.

[CR57] Saleh, Y. (2021). ICT, social media and COVID-19: Evidence from informal home-based business community in Kuwait City. *Journal of Enterprising Communities: People and Places in the Global Economy*, *15*(3), 395–413.

[CR58] Sein, M. K. (2020). The serendipitous impact of COVID-19 pandemic: A rare opportunity for research and practice. *International Journal of Information Management*, *55*, 102164. 32836630 10.1016/j.ijinfomgt.2020.102164 PMC7261442

[CR59] Sixsmith, A. (2020). COVID-19 and AgeTech. *Quality in Ageing and Older Adults*, *21*(4), 247–252.

[CR60] Sixsmith, A., Horst, B. R., Simeonov, D., & Mihailidis, A. (2022). Older people’s use of digital technology during the COVID-19 pandemic. *Bulletin of Science Technology & Society*, *42*(1–2), 19–24. 10.1177/02704676221094731 PMC9038938 38603230

[CR261] Tirado-Morueta, R., Aguaded-Gómez, J. I., & Hernando-Gómez, Á. (2018). The socio-demographic divide in internet usage moderated by digital literacy support. *Technology in Society, 55*, 47–55.

[CR61] United Nations Department of Economic and Social Affairs, Population Division. (2020). *World Population Ageing 2020 highlights: Living arrangements of older persons*. ST/ESA/SER.A/451.

[CR262] Ussher, Y. (2020). Exploring the effect of digital literacies on the use of mobile phones. In *Contemporary Issues in Human Resource Studies* (pp. 210–237). Woeli Publishing Services, Accra, Ghana.

[CR62] Yin, R. K. (2014). Case Study Research Design and methods. *Canadian Journal of Program Evaluation*, 1–5.

